# COVID-19 Perspectives of Assisted Living and Health Care Providers in Seven States

**DOI:** 10.1093/geroni/igab046.1042

**Published:** 2021-12-17

**Authors:** Sheryl Zimmerman, Philip Sloane, Johanna Hickey, Kali Thomas, Julia Thorp, Meredith Poole, Aisha Chaudhry, Paula Carder

**Affiliations:** 1 Cecil G. Sheps Center for Health Services Research, Chapel Hill, North Carolina, United States; 2 UNC Medical School, Sheps Center, Chapel Hill, North Carolina, United States; 3 University of North Carolina at Chapel Hill School, Chapel Hill, North Carolina, United States; 4 Brown University, Brown University/Providence, Rhode Island, United States; 5 OHSU-PSU School of Public Health, Portland, Oregon, United States

## Abstract

Thirty percent of COVID-19 deaths in long-term care were in assisted living (AL), indicating challenges providing care. This project recruited AL administrators and medical and mental health care providers in a seven-state stratified random sample of 250 communities; it asked what was most challenging responding to COVID-19, what was successful, how to have better dealt with COVID-19, and how others could have helped. The most common challenge was addressing residents’ psychosocial needs, explained as “No contact - no hugging. The seniors require touch. It's something we've always done, and we can't do; we're required not to do it.” Successes included infection prevention, and in hindsight, administrators discussed staffing. Related to external entities, one commented, “Come in the building and see what we're doing. Don't sit behind a freaking screen and act like you know what we're doing.” Providers stressed patient access to care and social isolation. Implications will be discussed.

